# Angiopoietin 1 release from human neutrophils is independent from neutrophil extracellular traps (NETs)

**DOI:** 10.1186/s12865-021-00442-8

**Published:** 2021-08-03

**Authors:** Elcha Charles, Benjamin L. Dumont, Steven Bonneau, Paul-Eduard Neagoe, Louis Villeneuve, Agnès Räkel, Michel White, Martin G. Sirois

**Affiliations:** 1 Research Center, Montreal Heart Institute, Université de Montréal, 5000 Belanger Street, Montreal, QC H1T 1C8 Canada; 2 Department of Pharmacology and Physiology , Université de Montréal, Montreal, QC Canada; 3 Department of Medicine, Université de Montréal, Montreal, QC Canada; 4 Faculty of Medicine, and Research Center—Centre Hospitalier de l’Université de Montréal (CHUM), Université de Montréal, Montreal, QC Canada

**Keywords:** Angiopoietin 1, Neutrophil, NETs, Heart failure, Type 2 diabetes, Calprotectin (S100A8/A9), Inflammation

## Abstract

**Background:**

Neutrophils induce the synthesis and release of angiopoietin 1 (Ang1), a cytosolic growth factor involved in angiogenesis and capable of inducing several pro-inflammatory activities in neutrophils. Neutrophils also synthesize and release neutrophil extracellular traps (NETs), comprised from decondensed nuclear DNA filaments carrying proteins such as neutrophil elastase (NE), myeloperoxidase (MPO), proteinase 3 (PR3) and calprotectin (S100A8/S100A9), which together, contribute to the innate immune response against pathogens (e.g., bacteria). NETs are involved in various pathological conditions through pro-inflammatory, pro-thrombotic and endothelial dysfunction effects and have recently been found in heart failure (HF) and type 2 diabetes (T2DM) patients. The aim of the present study was to investigate the role of NETs on the synthesis and release of Ang1 by the neutrophils in patients with T2DM and HF with preserved ejection fraction (HFpEF) (stable or acute decompensated; ADHFpEF) with or without T2DM.

**Results:**

Our data show that at basal level (PBS) and upon treatment with LPS, levels of NETs are slightly increased in patients suffering from T2DM, HFpEF ± T2DM and ADHF without (w/o) T2DM, whereas this increase was significant in ADHFpEF + T2DM patients compared to healthy control (HC) volunteers and ADHFpEF w/o T2DM. We also observed that treatments with PMA or A23187 increase the synthesis of Ang1 (from 150 to 250%) in HC and this effect is amplified in T2DM and in all cohorts of HF patients. Ang1 is completely released (100%) by neutrophils of all groups and does not bind to NETs as opposed to calprotectin.

**Conclusions:**

Our study suggests that severely ill patients with HFpEF and diabetes synthesize and release a greater abundance of NETs while Ang1 exocytosis is independent of NETs synthesis.

**Supplementary Information:**

The online version contains supplementary material available at 10.1186/s12865-021-00442-8.

## Background

Neutrophil extracellular traps (NETs) are composed of double-stranded DNA decorated with cytosolic and granule-derived pro-inflammatory cytokines and enzymes [1]. NETs are in response to inflammatory stimuli and carry cytoplasmic, granular and nuclear proteins (e.g. calprotectin; S100A8/A9, myeloperoxidase (MPO), neutrophil elastase (NE), histones and others) [2], contributing to the innate immune response against pathogens [1, 3, 4]. Although NETs were initially described as an antimicrobial mechanism of neutrophils and implicated in infectious disorders [1], other studies reported NETs involvement in the pathophysiology of non-infectious conditions such as thrombosis [5, 6], fibrosis [7, 8], inflammation [9] and cardiovascular disorders [10]. Even though there are common proteins linked to NETs, the variety and quantity of proteins bound to NETs can vary depending on the stimuli and pro-inflammatory conditions [11, 12].

Heart failure (HF) is a pro-inflammatory condition, in which the magnitude of inflammation is associated with the disease severity, being maximal in acute decompensated heart failure (ADHF) patients. Lately, we and other groups reported an increase of NETs formation (NETosis), either circulating or under in vitro neutrophil stimulation in type 2 diabetic (T2DM) patients [13–15] and in patients suffering from HF with or without (w/o) T2DM [15]. HF classification is based on left ventricular ejection fraction (LVEF), which can be reduced (HFrEF, LVEF ≤ 40%) or preserved (HFpEF, LVEF ≥ 50%), each with distinct phenotypes [16]. HFrEF is typically associated to primary myocardial lesion (e.g. myocardial infarction) leading to inadequate contractility of the left ventricle [16]. In contrast, HFpEF is a heterogeneous and multiorgan disorder, influenced by multiple comorbidities, including obesity, hypertension and T2DM. These conditions can lead to low-grade systemic inflammation, extensive endothelial and cardiac microvascular dysfunction, which can ultimately induce myocardial leukocytes migration, ventricular fibrosis, stiffening and dysfunction [17, 18]. In these conditions, impaired angiogenesis can occur and is mediated by growth factors such as vascular endothelial growth factor (VEGF) and angiopoietins (Ang). Over the last years, HFpEF prevalence outreached HFrEF cases, representing now > 50% of all HF patients [19, 20] and the attempts to transpose life-saving therapies from HFrEF to HFpEF have failed [21, 22]. It is therefore critical to find potential treatments for these patients.

There is also an increased incidence of ADHF, defined by a worsening of stable chronic HF [23]. This increases the rate of hospitalization and death in patients > 65 years old, who also have a 40% prevalence of T2DM [19]. The management of ADHF is different from stable HF [24], hence the importance of studying this pathology separately.

Calprotectin (S100A8/A9), a 36 kDa heterodimeric complex, is a cytosolic glycoprotein with two calcium binding of the S100 protein family and is constitutively expressed in neutrophils, monocytes, and macrophages [25–28]. Calprotectin, known for its antimicrobial functions [27, 28], is carried by NETs [3] and used as an inflammatory marker in the diagnostic of non-infectious inflammatory disorders such as arthritis, bowel [29, 30] and cardiovascular diseases (e.g. myocardial infarction, unstable angina and chronic HF) [31]. Angiopoietin 1 (Ang1) is a secreted 70-kDa glycoprotein constitutively expressed by vascular smooth muscle cells [32], platelets [33], pericytes, monocytes and neutrophils [34, 35] and a key regulator for angiogenesis, through vascular stabilisation and maturation [36]. Ang1 may also play a role in endothelial dysfunction associated with cardiovascular diseases such as HF and T2DM [37, 38]. Yet, it is unknow if the release of Ang1 by the neutrophils is associated to NETs synthesis and release.

The objective of this study was to determine the capacity of various inflammatory mediators (LPS, PMA and A23187) to induce NETs, Ang1 and calprotectin synthesis and release, and if Ang1 can bind to NETs, using calprotectin as a positive control, from neutrophils of patients with stable or decompensated HF with or w/o T2DM compared with healthy control (HC) volunteers.

## Results

The clinical characteristics of the study population are presented in Table 1. The study population consisted of 34 healthy control (HC) volunteers, 8 patients with T2DM and without HF-pEF, 12 patients with HFpEF and with stable symptoms (7 with T2DM) and 13 patients with ADHFpEF (6 with T2DM). Most HF and ADHF patients had a HF caused by cardiomyopathy. All patients with stable HF and a majority of patients with ADHF + T2DM suffered from hypertension. There was no significant difference in LV ejection fraction between stable or ADHF patients with or without diabetes. All T2DM patients were treated with statins. The majority (> 70%) of stable HF or ADHF patients were treated by oral anticoagulants.

**Table 1:** Baseline patients characteristics

	HC (n = 34)	T2DM group (n = 8)	Stable HFpEF (n = 5)	Stable HFpEF + T2DM (n = 7)	ADHFpEF (n = 7)	ADHFpEF + T2DM (n = 6)
Age (years)		68 ± 1.7	75 ± 3.3	71 ± 3.2	82 ± 3.0*	73 ± 3.5
Males *n* (%)	20 (58.8%)	6 (75%)	2 (40%)	4 (57.1%)	2 (28.6%)	4 (66.7%)
*NYHA classification n (%)*						
Class I	n/a	n/a	0 (0%)	1 (14.3%)	0 (0%)	0 (0%)
Class II	n/a	n/a	3 (60%)	5 (71.4%)	2 (28.6%)	1 (16.7%)
Class III	n/a	n/a	2 (40%)	1 (14.3%)	2 (28.6%)	2 (33.3%)
Class IV	n/a	n/a	0 (0%)	0 (0%)	2 (28.6%)	2 (33.3%)
*Etiology n (%)*						
Ischemia	n/a	n/a	0 (0%)	1 (14.3%)	0 (0%)	2 (33.3%)
Cardiomyopathy	n/a	n/a	1 (20%)	5 (71.4%)	3 (42.9%)	1 (16.7%)
Valvular	n/a	n/a	0 (0%)	3 (42.9%)	1 (14.3%)	2 (33.3%)
Others	n/a	n/a	4 (80%)	0 (0%)	4 (57.1%)	0 (%)
LVEF (%)	n/a	n/a	54 ± 2.1	58 ± 1.5	57 ± 2.6	55 ± 1.3
Hypertension	n/a	6 (75%)	5 (100%)	7 (100%)	4 (57.1%)	6 (100%)
Dyslipidemia	n/a	7 (87.5%)	1 (20%)	4 (57.1%)	3 (42.9%)	5 (83.3%)
Stroke	n/a	0 (0.0%)	0 (0.0%)	0 (0.0%)	0 (0.0%)	0 (0.0%)
*Biochemistry*						
Creatinine (μmol/l)	n/a	86.7 ± 8.6	147 ± 43	146 ± 17	127 ± 21	166 ± 26
Glucose (mmol/l)	n/a	9.0 ± 0.7	5.7 ± 0.6*	6.9 ± 0.6	6.4 ± 0.6	8.0 ± 0.6
Triglyceride (mmol/l)	n/a	1.35 ± 0.19	1.02 ± 0.12	2.06 ± 0.56		1.43 ± 0.13
LDL (mmol/l)	n/a	1.80 ± 0.12	2.23 ± 0.80	1.09 ± 0.23		1.64 ± 0.50
*Medication n (%)*						
ACEi	n/a	3 (37.5%)	0 (0.0%)	1 (14.3%)	0 (0.0%)	1 (16.7%)
ARBs	n/a	5 (62.5%)	0 (0.0%)	2 (28.6%)	1 (14.3%)	0 (0.0%)
β-blockers	n/a	3 (37.5%)	3 (60%)	5 (71.4%)	5 (71.4%)	5 (83.3%)
Diuretic agents	n/a	2 (25%)	5 (100%)	7 (100%)	5 (71.4%)	6 (100%)
Statins	n/a	8 (100%)	2 (40%)	3 (42.9%)	2 (28.6%)	5 (83.3%)
Anticoagulants	n/a	0 (0.0%)	5 (100%)	4 (57.1%)	6 (85.7%)	3 (50.0%)
Sulfonylureas	n/a	3 (37.5%)	0 (0.0%)	1 (14.3%)	0 (0.0%)	2 (33.3%)
DDP-4 inhibitors	n/a	5 (62.5%)	0 (0.0%)	4 (57.1%)	0 (0.0%)	1 (16.7%)
GLP-1 agonists	n/a	2 (25%)	0 (0.0%)	0 (0.0%)	0 (0.0%)	0 (0.0%)
SGLT-2 inhibitors	n/a	2 (25%)	0 (0.0%)	2 (28.6%)	0 (0.0%)	1 (16.7%)
Metformin	n/a	7 (87.5%)	1 (20%)	4 (57.1%)	0 (0.0%)	1 (16.7%)
Insulin	n/a	1 (12.5%)	0 (0.0%)	2 (28.6%)	0 (0.0%)	2 (33.3%)

HC, healthy controls; HFpEF, heart failure with preserved ejection fraction; ADHF, acute decompensated heart failure; T2DM, type-2 diabetes mellitus; NYHA, New York Health Association; LVEF, left ventricular ejection fraction; ACEi, angiotensin-converting enzyme inhibitor; ARB, angiotensin receptor blocker; DPP-4, dipeptidyl peptidase 4; GLP-1, glucagon-like peptide 1; SGLT2, sodium-glucose co-transporter-2. Continuous variables are shown as mean ± standard error mean and categorical variables as number (%). Anti-platelets included aspirin,clopidogrel, prasugrel, or ticagrelor or a combination of these. *p < 0.05 versus T2DM

### NETs release by neutrophils

**Fig. 1:**
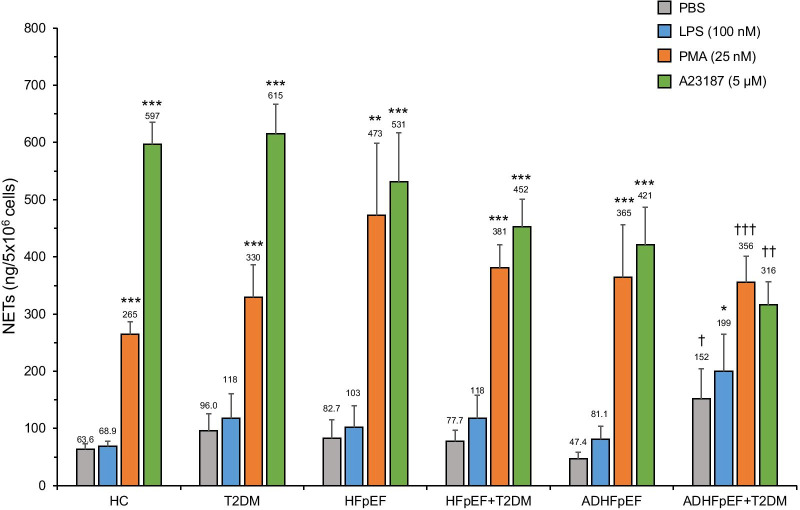
NETs release by neutrophils. Isolated neutrophils (5 × 10^6^/mL) from HC, T2DM, stable HFpEF ± T2DM and ADHFpEF ± T2DM patients were incubated at 37 °C for 3 h with PBS (control vehicle) and agonists, LPS (100 nM), PMA (25 nM), or A23187 (5 μM). NETs were quantified using Quant-IT PicoGreen dsDNA detection kit. Data shown as mean ± SEM. Significance of data is indicated by *p < 0.05, **p < 0.01, and ***p < 0.001 compared with PBS respectively and by ^†^*p* < 0.05, ^††^*p* < 0.01, ^†††^*p* < 0.001 compared with corresponding treatment of HC. HC (n = 28–32), T2D (n = 8), stable HFpEF (n = 5), stable HFpEF + T2DM (n = 6), ADHF (n = 6–7), ADHF + T2DM (n = 5–6)

The rate of NETs synthesis and release for all 6 cohorts (HC, T2DM, stable HFpEF ± T2DM and ADHFpEF ± T2DM patients) is presented in Fig. 1. The basal value of NETs (quantified as dsDNA) released by neutrophils from HC treated with PBS (basal control) for 3 h was 63.6 ± 9.6 ng/5 × 10^6^ neutrophils/mL (Fig. 1). In HC, we observed a significant 4.2-fold (265 vs 63.6 ng/mL) and 9.4-fold (597 vs 63.6 ng/mL) NETs increase post-stimulation with PMA and A23187 respectively compared to PBS, whereas LPS did not increase NETs release. A similar pattern was observed in PMA- and A23187-stimulated neutrophils from T2DM patients, stable HFpEF ± T2DM and ADHFpEF without T2DM (Fig. 1). Basal and LPS-stimulated neutrophils from ADHFpEF + T2DM patients released significantly more NETs (2.4-fold (152 vs 63.6 ng/mL) and 2.9-fold (199 vs 63.6 ng/mL) respectively), while the A23187 stimulation released a significant lower quantity of NETs (47% decrease; 316 vs 597 ng/mL) compared to HC. When comparing ADHF w/o T2DM versus ADHF + T2DM, we observed that NETs release increased in basal condition (3.2-fold; 152 vs 47.4 ng/mL) and LPS-stimulated neutrophils (2.5-fold; 199 vs 81.1 ng/mL).

### Angiopoietin 1 release, intracellular content and NETs binding in neutrophils

**Fig. 2:**
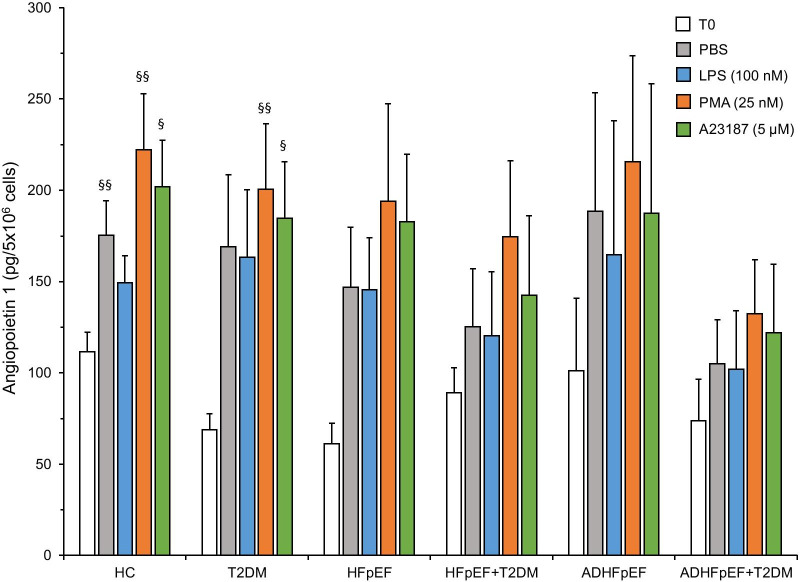
Angiopoietin 1 release from neutrophils. Isolated neutrophils (5 × 10^6^/mL) from HC, T2DM, stable HFpEF ± T2DM and ADHFpEF ± T2DM patients were incubated at 37 °C for 3 h with different agonists (PBS control, PMA, LPS and A23187). By ELISA, we quantified Ang1 from unstimulated neutrophils (T0) and stimulated neutrophils. ^§^*p* < 0.05 and ^§§^*p* < 0.01 versus T0

We assessed the Ang1 release, intracellular content and binding capacity to released NETs in isolated neutrophils from all 6 cohorts upon stimulation with PBS, LPS, PMA and A23187 for 3 h. The post-isolation intracellular content of Ang1 (T0) was 112 ± 11 pg/5 × 10^6^ neutrophils/mL from HC. There was a non-significant decrease in Ang1 concentrations at T0 from neutrophils of all five patients’ cohorts (Fig. 2). After a 3-h treatment with PBS, LPS, PMA or A23187, the Ang1 content released by neutrophils from all cohorts was higher than before treatment (T0), but only significant in PBS-(1.56-fold; 175 vs 112 pg/mL), PMA-(1.98-fold; 222 vs 112 pg/mL) and A23187-(1.80-fold; 202 vs 112 pg/mL) stimulated neutrophils from HC. The concentration of Ang1 detected intracellularly or bound to NETs following a 3-h stimulation with all agonists in all 6 cohorts were below the lower limit of quantitation (LLOQ; < 156 pg/mL), and extrapolated from the ELISA standard curve. Therefore, the total amount of Ang1 detected at 3 h post-treatment was considered as being almost completely released, indicating a significant increase of Ang1 synthesis (up to 1.98-fold; 202 vs 112 pg/mL) in neutrophils from HC, and this effect was amplified in PMA and A23187 stimulated neutrophils from T2DM patients (up to 2.91-fold; 201 vs 69 pg/mL). In all other cohorts, independently from the agonists used, Ang1 synthesis of also increased (up to 3.18-fold; 216 vs 101 pg/mL in HFpEF PMA-stimulated neutrophils) (Fig. 2).

### Calprotectin release, intracellular content and NETs binding in neutrophils

**Fig. 3:**
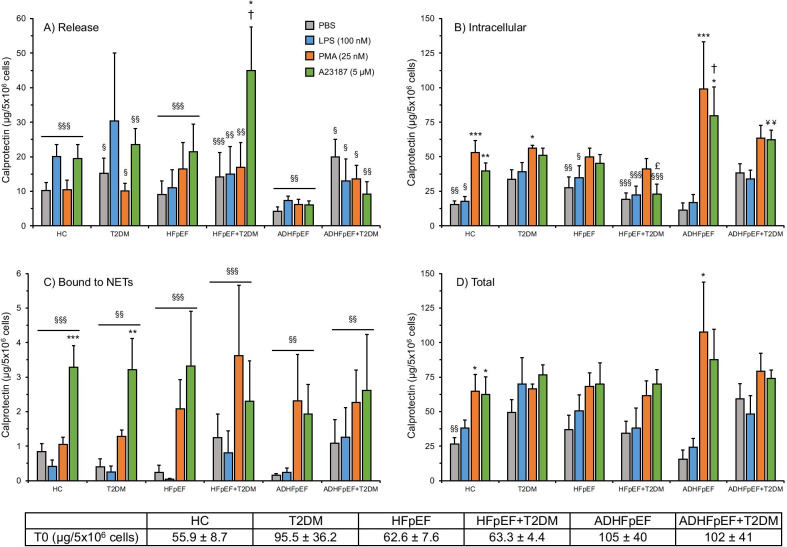
Calprotectin synthesis and release from neutrophils. Isolated neutrophils (5 × 10^6^/ml) from HC, T2DM, stable HFpEF ± T2DM and ADHFpEF ± T2DM patients were incubated at 37 °C for 3 h with different agonists (PBS control, PMA, LPS and A23187). By ELISA, we quantified calprotectin from unstimulated (T0) and stimulated neutrophils. Total calprotectin corresponds to the addition of calprotectin released, intracellular and bound to NETs. ^§^*p* < 0.05, ^§§^*p* < 0.01 and ^§§§^*p* < 0.001 versus T0, *p < 0.05, **p < 0.01 and ***p < 0.001 versus PBS, ^†^*p* < 0.05 versus HC-corresponding agonist, ^£^*p* < 0.05 versus T2DM-corresponding agonist and ^¥¥^*p* < 0.01 versus HFpEF + T2DM

We assessed the calprotectin (S100A8/S100A9) release, intracellular content and binding capacity to released NETs in isolated neutrophils from all 6 cohorts upon stimulation with PBS, LPS, PMA, and A23187 for 3 h. The post-isolation calprotectin intracellular content (T0) in neutrophils from HC was 55.9 ± 8.7 µg/5 × 10^6^ neutrophils/mL, while a higher concentration (non-significant) was observed for all patient’s cohorts (Fig. 3).

In all 6 cohorts and independently from the agonist used, the calprotectin released was significantly lower than the initial quantity found in post-isolation neutrophils (T0). None of the agonists significantly increased the calprotectin release when compared to unstimulated neutrophils (PBS) (Fig. 3A).

In all 6 cohorts and independently from the agonist used, the intracellular calprotectin was lower than the initial quantity found in post-isolation neutrophils (T0). PMA-stimulated neutrophils from HC, T2DM and ADHFpEF increased significantly (up to 8.8-fold; 99 vs 11 µg /mL in ADHFpEF) the calprotectin intracellular content compared to PBS. A23187-stimulated neutrophils from HC significantly increased the intracellular calprotectin (2.6-fold; 39.6 vs 15.5 µg/mL), HFpEF + T2DM had a significantly lower concentration of intracellular calprotectin (55% decrease; 22.8 vs 51.1 µg/mL) compared to T2DM patients, whereas in ADHFpEF patients it increased significantly (2.0-fold; 79.5 vs 39.6 µg/mL) compared to HC. Finally, we observed a significant increase (2.7-fold; 62.3 vs 22.8 µg/mL) in the calprotectin neutrophil content from ADHFpEF + T2DM compared to HFpEF + T2DM (Fig. 3B).

In all 6 cohorts and independently from the agonist used, calprotectin was detected on NETs but was significantly lower than in the intracellular fraction post-isolation (T0). A treatment with PMA increased the calprotectin NETs binding in all cohorts by up to 15-fold in ADHFpEF (2.30 vs 0.15 µg/mL), while the A23187 stimulation provided a significant increase in HC and T2DM (up to 8-fold; 3.20 vs 0.40 µg/mL in T2DM) compared to PBS (Fig. 3C).

The total calprotectin (released + intracellular + bound to NETs) following the 3-h neutrophil incubation with PBS or LPS was lower than post-isolation (T0) in all 6 cohorts, but only significant for PBS in HC (53% decrease; 26.5 vs 55.9 µg/mL). When stimulated with PMA for 3 h, the total calprotectin remained unchanged compared to T0, while being significantly higher in HC (2.4-fold; 64.7 vs 26.5 µg/mL) and in ADHFpEF (6.9-fold; 108 vs 15.6 µg/mL) when compared to PBS (Fig. 3C).

### Imaging of Ang 1 and calprotectin localization in human neutrophils

**Fig. 4:**
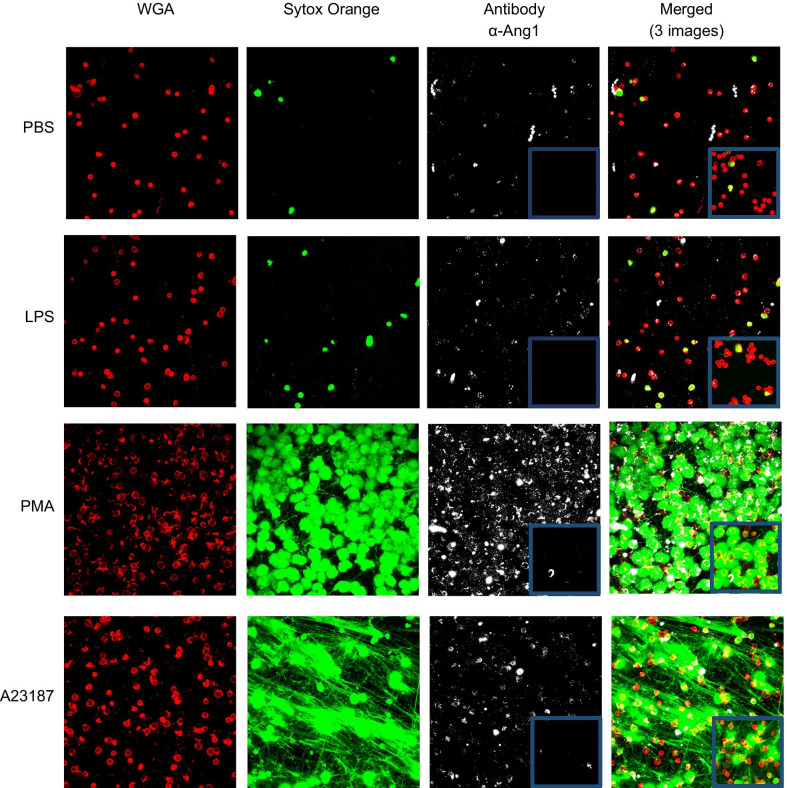
Ang1 is released independently from NETs. Neutrophils from HC subjects were stimulated with PBS, LPS (100 nM), PMA (25 nM), or A23187 (5 μM) for 3 h to induce NETosis. Following stimulation, neutrophils were incubated with a primary unconjugated Ab (Rabbit α-human Ang1), followed by an incubation with a conjugated secondary Ab (Alexa Fluor 488 goat anti-rabbit; white). For the negative control, neutrophils were incubated only with the secondary Ab (blue boxes). Neutrophils were labeled with wheat germ agglutinin (conjugated with Alexa 647; red) and NETs were labeled with SYTOX Orange (green pseudo-color). Maximum intensity projection from acquired Z-stack were obtained by confocal microscopy (LSM 710, Carl Zeiss) using a Plan Apochromat 40x/1.3 oil DIC objective

**Fig. 5:**
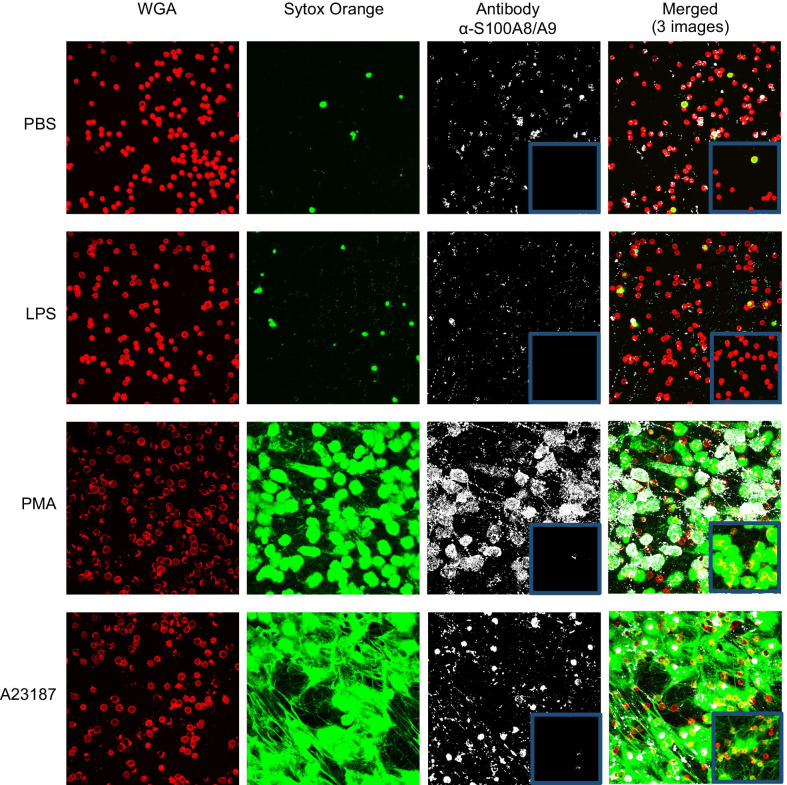
Calprotectin (S100A8/A9) binds to NETs. Neutrophils from HC subjects were stimulated with PBS, LPS (100 nM), PMA (25 nM), or A23187 (5 μM) for 3 h to induce NETosis. Following stimulation, neutrophils were incubated with a primary unconjugated Ab (mouse α-S100A8/A9), followed by an incubation with a conjugated secondary Ab (Alexa Fluor 488 anti-mouse; white). For the negative control, neutrophils were incubated only with the secondary Ab (blue boxes). Neutrophils were labeled with wheat germ agglutinin (conjugated with Alexa 647; red) and NETs were labeled with SYTOX Orange (green pseudo-color). Maximum intensity projection from acquired Z-stack were obtained by confocal microscopy (LSM 710, Carl Zeiss) using a Plan Apochromat 40x/1.3 oil DIC objective

Based on our aforementioned data, we sought to visualize whether Ang1 and calprotectin behave differently in regard to their binding to NETs, using confocal microscopy. Neutrophils isolated from HC were treated with PBS, LPS, PMA, and A23187 for 3 h, followed by a series of incubations with antibodies detecting either Ang1 (Fig. 4) or calprotectin (Fig. 5).

**Fig. 6:**
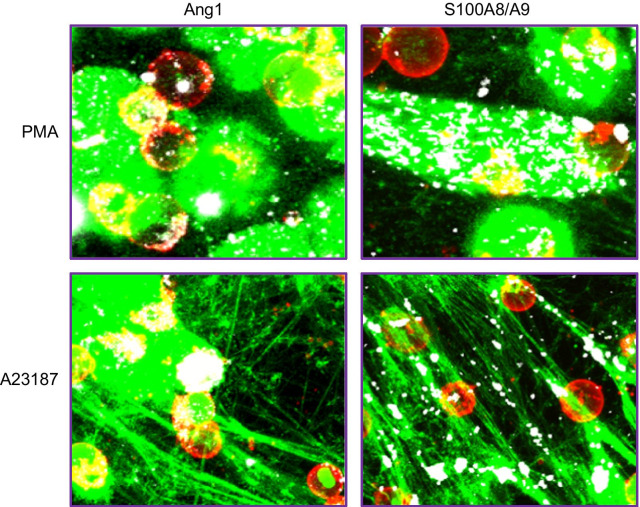
Ang1 and calprotectin interaction with NETs induced by PMA and A23187. These images are enlarged areas of the “Merged (3 images)” columns (PMA and A23187) from Figs. 2 (Ang1) and 3 (calprotectin). We observe that Ang1 is mainly detected around the cells surface and does not interact with NETs, whereas calprotectin is detected either in the cells or on the NETs web-like structures

First, we observed that in unstimulated neutrophils (PBS), there is no or marginal detection of SYTOX Orange (green pseudo-color) due to a very low percentage of permeabilized cell membrane and absence of intracellular DNA exposure. Treatment with LPS induced a small synthesis and release of NETs (green pseudo-color), whereas a treatment with PMA and A23187 induced a marked increase of NETs synthesis and release (Figs. 4 and 5; SYTOX Orange column). For the detection of Ang1 and calprotectin proteins, we observed a similar pattern, namely a marginal intracellular detection of both proteins under PBS and LPS treatments, whereas under PMA and A23187 stimulation, cell permeabilization associated to NETosis allowed the specific binding of Ang1 and calprotectin antibodies intracellularly (Figs. 4 and 5; Antibody and merged columns). In addition to the detection of Ang1, calprotectin and NETs within the neutrophils, we observed that Ang1 as opposed to calprotectin does not seem to be bound to extracellular NETs (Fig. 6).

## Discussion

In the present study, we report that neutrophils from patients diagnosed with T2DM alone, HFpEF ± T2DM or ADHFpEF w/o T2DM present a slight non-significant NETs increase, whereas ADHFpEF + T2DM patients have a higher significant NETs release after a 3-h incubation with PBS (control vehicle) or LPS (a weak NETs inducer), compared to HC. In addition, ADHFpEF + T2DM patients present a lower Ang1 release from their neutrophils, while having a higher capacity to promote calprotectin release under basal (PBS) condition. Finally, in all 6 cohorts and independently from the agonist used, we observed that, while calprotectin was found to bind to the NETs web-like structures, Ang1 did not interact with NETs, suggesting that NETs are selective transporters of proteins. These data suggest that patients with symptomatic HFpEF exhibit significant neutrophil activation and NETs release. The magnitude of NETs release is significantly increased in patients with ADHF.

### NETs release from human neutrophils

It has been demonstrated that neutrophils are not just first responders to acute infections but also active contributors to low-grade chronic inflammation [39], which can be explained, in part, by their capacity to release NETs [40]. NETs can be considered as a risk factor of future cardiovascular events because of their role in atherosclerosis, inflammation, and vascular thrombosis [2, 10, 41, 42]. There has been little precious data on the release of NETs in the context of heart failure. More recently, we and other groups reported elevated levels of circulating NETs in T2DM patients and an increase in their neutrophil capacity in vitro to release NETs [14, 15, 43]. Furthermore, we observed an increase in circulating NETs and corresponding release in HF patients, the latter reaching a maximum in HF + T2DM [15]. The results from this study confirm that NETs release is significantly increased in clinical HF and more so in patients with HF and T2DM. In this study, we used inflammatory mediators, such as LPS, PMA and A23187, targeting different signalling pathways to induce NETosis by human neutrophils [44–48]. LPS, a component of gram-negative bacteria, induces NADPH oxidase (NOX)-dependent NETs formation mediated by c-Jun N-terminal kinases (JNK). LPS binds to Toll-like receptor (TLR4) on the surface of neutrophils, activating the production of reactive oxygen species (ROS) and NOX, inducing lytic NETs formation in a concentration- and NOX-dependent manner [46]. Other groups have shown that LPS can also induce vital NETs formation via a NOX-independent pathway [47, 48]. PMA activates the protein kinase C pathway, which induces NETosis through the ROS generating NADPH oxidase complex that contributes to the disruption of the extracellular membrane. In contrast, A23187, is a faster and robust, NADPH-independent, process dominated by a rise in intracellular calcium concentration [45, 49]. This might explain why the elevation of NETs synthesis under PMA is similarly maintained in all groups, whereas we observed a reduction of NETosis in A23187-stimulated neutrophils from AFDHFpEF + T2DM patients. Since the neutrophils from these patients are as responsive as the neutrophils from other groups under PMA stimulation, this might suggest that their stimulation with a calcium ionophore (A23187) is less efficient to promote extracellular Ca^2+^ uptake and/or intracellular Ca^2+^ elevation affecting the downstream NETosis process.

In the present study, we sought to determine the ability of these inflammatory mediators to induce NETs formation by neutrophils from HC, T2DM alone, stable HFpEF ± T2DM and ADHFpEF ± T2DM patients. Our study revealed significantly higher basal (PBS) and LPS-induced NETs release only in patients with ADHFpEF + T2DM, as compared with HC. In addition, released NETs were increased at basal level and significantly following LPS stimulation in ADHFpEF + T2DM patients versus ADHFpEF w/o T2DM, suggesting that T2DM contributes to increase the inflammatory state in ADHFpEF patients. We did observe an increase, although non-significant, in basal or LPS-induced NETs release in T2DM alone or stable HFpEF ± T2DM patients, and this might be an indicator that the management of their low-grade inflammatory condition by the current chronic therapies is not fully capable to revert the inflammatory state associated to NETosis.

The increase in NETs release seen in ADHFpEF + T2DM patients support the concept that these patients are in a state of acute thrombo-inflammation. Such status may lead to a pro-thrombotic state wherein blood vessel obstruction could result in inadequate blood supply to the heart and/or other organs. Therefore, NETs and T2DM can jointly contribute to the progression and severity of HF, leading ultimately to the ADHF condition.

### Angiopoietin 1 release and NETs binding

In this study, we wanted to determine if the release of Ang1 was comparable to calprotectin, since both proteins are found in the cytosol of neutrophils. We previously demonstrated that in healthy controls, Ang1 is found in the cytosol of neutrophils and can be released upon stimulation with different stimuli [35]. Ang1 is an important inflammatory marker for the stabilization and maturation of blood vessels through Tie2 receptor [34, 50]. Studies have shown that a decrease of circulating Ang1 levels in patients with acute myocardial infarction and major cardiovascular conditions such as arrhythmia, valvular heart disease, HF and cardiogenic shock, could be potentially associated with the magnitude of endothelial dysfunction [37, 51]. In our study, we also observed a lower concentration of intracellular Ang1 in post-isolated (T0) neutrophils in all patients’ cohorts. These neutrophils increase their Ang1 synthesis over a 3-h incubation time period, yet, their corresponding Ang1 concentrations never reached the levels observed in HC. Interestingly, the lowest Ang1 concentration post-incubation was observed in both ADHFpEF + T2DM and stable HFpEF + T2DM suggesting that T2DM co-morbidity is negatively impacting the capacity of neutrophils from HF patients to synthesize Ang1. This could contribute to inadequate stabilization of blood vessels, endothelial dysfunction and disease progression.

Since neither calprotectin nor Ang1 are stored in neutrophil granules or vesicles [35, 52, 53], and that calprotectin binds to NETs when released, we wanted to assess if Ang1 exocytosis was also NETs-dependent. Herein, we observed that Ang1 was not detected on the surface of extracellular NETs, either by ELISA or using confocal microscopy, suggesting that its secretion is NETs-independent in all 6 cohorts studied. This could be explained by the fact that Ang1 is not an essential bactericidal protein, like MPO, NE or calprotectin, all found on NETs. Moreover, Ang1 has been shown to bind either to Tie2 receptor and selected integrins, both expressed on cell membrane surface of neutrophils [7, 54, 55]. In addition, the low concentration of Ang1 (~ 100–200 pg/5 × 10^6^ neutrophils) being released as compared to calprotectin (~ 5–20 µg/5 × 10^6^ neutrophils) could explain their membrane proximity, as observed by confocal microscopy, suggesting an autocrine agonistic activity upon its release from the neutrophils.

### Calprotectin release and NETs binding

Calprotectin (S100A8/A9) is largely expressed in the cytoplasm of neutrophils and is mainly released in an infectious setting. Moreover, calprotectin can bind to NETs [3] and exerts its main function, namely anti-microbial, in combination with other NETs-bound proteins such as MPO and NE, while NETs are keeping pathogens trapped, thus leading to increased efficiency in pathogens removal.

Calprotectin has also recently been shown to be involved in cardiovascular diseases, following its release by inflammatory mediators [56, 57]. Previous studies reported a higher serum or plasma calprotectin level in patients with chronic HF and was associated with other inflammatory markers such as C-reaction protein (CRP), interleukin IL-6, IL-8 and TNF-α [31, 58, 59]. In the present study, we observed an increase of intracellular calprotectin in neutrophils post-isolation (T0) from T2DM and ADHFpEF ± T2DM patients as compared to HC, whereas in stable HFpEF ± T2DM the calprotectin initial content remained unchanged. However, there was no additional calprotectin synthesis after 3 h of stimulation in all 6 cohorts and independently from the agonists used. The increase of endogenous calprotectin observed in freshly isolated neutrophils from these patients could indicate an increased inflammatory status, thereby contributing to the progression of heart failure.

The levels of released calprotectin were significantly lower than those seen at T0, but interestingly, there was less calprotectin released from the neutrophils of patients with T2DM and HFpEF + T2DM after treatment with PMA contrary to what was observed after stimulation with A23187. Typically, A23187 induces a rapid and robust extracellular DNA release, reaching a plateau within 3–4 h, whereas NETs formation induced by PMA is slower and reaching maximal extracellular DNA after 4–6 h [49]. Since calprotectin is present in high concentration in the cytosol but not in the granules, treatment with a calcium ionophore might favor its higher release compared to PMA stimuli in the neutrophils from the patients.

After 3 h of stimulation and in absence of agonist stimulation (PBS), we observed in all 6 cohorts that the level of calprotectin was reduced by 40 to 85% compared to corresponding T0 values. In addition, even after stimulation with LPS, PMA or A23187 agonists, the levels of calprotectin remained either below or comparable to T0 values. Thus, we hypothesized that this decrease could be associated to calprotectin degradation by the proteasome. However, when using the proteasome inhibitor (MG132; 10 µM) [60], it did not prevent the reduction of intracellular calprotectin concentration, neither the total concentration of calprotectin (data not shown). One possibility might be that oxidative post-translational modifications of calprotectin makes it a target for proteasome-independent proteolysis [61].

As previously described [1, 3], we observed that a fraction (up to 5.3%) of the total calprotectin detected at 3 h post-incubation was bound to NETs as observed by ELISA and confocal microscopy (Figs. 3 and 5). Only NETs produced by PMA and A23187 bound more calprotectin, since those two agonists induced a higher NETs release in all cohorts (Fig. 1). Interestingly, in HC and T2DM patients, A23187 induced the release of mostly web-like NETs as seen in Fig. 3, and this conformation bound more calprotectin than the PMA-mediated NETs synthesis, localized mostly near the cells. This could be explained by the fact that NETs web-like structures are observed in microbial trapping, thus supporting the calprotectin antimicrobial role [1, 62, 63].

### Study limitations

This study consisted of a small sample size of patients with various duration, etiology and severity of HF. Clinically relevant information such as the duration of diabetes, glycemic control, and the concomitant presence of atherosclerotic heart disease were not readily available. In vitro neutrophil experiments were limited to stimulation with few agonists and to the measure of Ang1 and calprotectin. Future studies warrant the inclusion of other cytokines and higher number of patients.

## Conclusions

In our study, NETs released by isolated neutrophils upon stimulation with selected agonists were significantly increased in ADHFpEF + T2DM when compared to healthy control volunteers. In addition, the release of Ang1 is independent from NETosis and not affected by diabetes or heart failure conditions. On the other side and as expected, calprotectin does bind to NETs, with the constitutive basal levels of calprotectin tending to increase in neutrophils from T2DM and ADHFpEF ± T2DM patients. Since neutrophils from ADHF + T2DM have a higher capacity to release NETs under basal condition, their capacity to bind calprotectin might further exacerbate NETs-mediated pro-inflammatory activities in these patients.

## Methods

### Population

This was a prospective non-randomized non-interventional study including stable HFpEF or ADHFpEF, with or without T2DM, compared with T2DM patients and HC without any heart pathology. Six different cohorts were recruited at the Montreal Heart Institute (MHI): (1) HC (n = 34), (2) T2DM (n = 8), (3) stable HFpEF (n = 5), (4) stable HFpEF + T2DM (n = 7), (5) ADHFpEF (n = 7) and (6) ADHFpEF + T2D (n = 6). Blood collection from all participants was performed at the MHI. The study has been approved by the MHI’s Research Ethics Committee and performed with the accordance of the Declaration of Helsinki. Informed consent was obtained from all subjects prior to the study (Montreal, QC, Canada; ethics No. ICM#01-069 and No. ICM #12-1374).

### Selection criteria of healthy control volunteers and patients

Healthy controls (HC) recruited in this study were enrolled assuming they had no significant medical conditions and were not treated by any anti-inflammatory medication for at least 14 days before blood collection. T2DM patients with no symptoms or signs of HF were recruited from the *Clinique d'Endocrinologie de Montréal*. HFpEF and ADHFpEF patients with NYHA classification I to IV symptoms were recruited from the MHI heart failure clinic and from the emergency room (ER) or HF care units respectively. These patients were classified as HFpEF if their LVEF was ≥ 50% [64–66], as documented by contrast ventriculography, magnetic resonance imaging, radionuclide ventriculography or echocardiography assessed within the previous 12 months and if no significant cardiac events occurred since the assessment of LVEF [65]. These patients were optimally treated on stable doses of A-II modulating agents, beta-blocker, and mineralo-corticoid antagonist agents unless not tolerated or contra-indicated. In addition to the previous inclusion criteria outlined above, patients with HF + T2DM required an HbA1c < 10% and good glycemic control by any available hypoglycaemic medications and treated with secondary preventive medication as per current guidelines. The most significant exclusion criteria included the presence of severe chronic pulmonary disease, chronic active inflammatory disease, severe renal failure (creatinine > 250 µmol/L), liver damage (transaminases ≥ threefold upper normal values) and ongoing malignancy. Other exclusion criteria included recent myocardial infarction, stroke, or cardiac surgery (< 3 months). All participants having ongoing and/or recent infection within 2 weeks prior to the study were excluded from this study.

### Study protocol—plasma, serum and neutrophil collection

Venous blood from all participants was collected in 30 mL syringes (containing 5 mL acid citrate dextrose for 25 mL whole blood). Neutrophils were isolated using the Ficoll-Paque gradient method, as previously described [67, 68]. Upon isolation, neutrophils were resuspended in phenol-free RPMI-1640 medium (Cambrex Bio Science, Walkersville, MD) supplemented with (1) 25 mM HEPES (N-2-hydroxyethylpiperazine-N′-2-ethanesulfonic acid) (Sigma-Aldrich, Oakville, ON, Canada), (2) 1% penicillin/streptomycin/ Glutamax (VWR Intl., Montreal, QC, Canada), (3) 1 mM CaCl_2_ (BDH Chemicals, Toronto, ON, Canada) and (4) 5% FBS (Fetal Bovine serum; VWR) (termed complete RPMI). Contamination by PMBCs was less than 0.1% as determined by morphological analysis and flow cytometry. Cell viability of neutrophils were greater than 98%, as assessed by Trypan blue dye exclusion assay.

### NETs production and quantification by fluorometric assays

Isolated neutrophils (5 × 10^6^/ml) were added to 12-well plates and incubated in complete RPMI at 37 °C for 3 h with either PBS-control buffer, LPS (100 nM; Escherichia coli O111:B4; Sigma), PMA (25 nM; Calbiochem, La Jolla, CA, USA) or A23187 (5 μM; Calbiochem). Neutrophils were carefully washed two times with PBS 1X, and nuclease S7 (Sigma) was added for 15 min at 37 °C, 5% CO_2_ to release NETs bound to the external surface of neutrophils with no or minor loss of NET structure and activity. The reaction was stopped with 10 mM EDTA (Sigma), and the supernatant was centrifuged at 300 g for 5 min to remove cell debris. NETs were quantified using Quant-IT PicoGreen dsDNA Assay Kits (catalog no. P7589; Invitrogen, Eugene, OR).

### Localisation and release of Ang1 and calprotectin by ELISA

The intracellular Ang1 and calprotectin concentrations in neutrophils (5 × 10^6^ cells/mL) were determined either immediately after isolation (T = 0), or upon agonists (PBS, LPS, PMA or A23187) stimulation in 6-well plates for 3 h at 37 °C, 5% CO_2_. The supernatants were collected and centrifuged at 300 g for 5 min to remove cell debris. Neutrophils were then carefully washed two times with PBS, and DNase I (Sigma) was added for 30 min at 37 °C, 5% CO_2_. The supernatant was collected in 10 mM EDTA to stop the reaction and centrifuged at 300 g for 5 min to remove cell debris. A solution containing complete RPMI + 1% Triton was added to the remaining adhered neutrophils which were then removed using a cell lifter, homogenized by vortex mixing and centrifuged at 18,000*g* for 10 min. The cell membranes pellet was discarded and the supernatant was used for intracellular content measures. All samples were stored at − 80 °C for further Ang1 quantification using ELISA DuoSet kits (R&D System). Since the concentration of Ang1 was too low for direct detection by ELISA, all samples were concentrated fivefold by evaporating all the water (1 mL) from the samples using a SpeedVac and resuspending the dry fraction in 200 µL of complete RPMI prior to their quantification.

### Localisation of calprotectin and Ang1 on NETs by confocal microscopy

Neutrophils (1 × 10^6^/mL) in complete RPMI were incubated in 35 mm petri dishes with 14 mm microwell insert (MatTek; #P35G-1.0-14-C Ashland, MA, USA) for 3 h at 37 °C, 5% CO_2_ with different agonists (PBS, PMA, LPS and A23187). After carefully removing the supernatant, 1% BSA (Bovine serum albumin) was added for 30 min at 37 °C, 5% CO_2_. Primary antibodies (rabbit anti-human Ang1 (1:100) and mouse anti-human S100A8/A9 (1:20)), IgG isotype control (rabbit or mouse (both 1:200)) were added directly for 30 min at 37 °C, 5% CO_2_, followed by a gentle wash with HBSS 1X. Subsequently, secondary antibodies (Alexa Fluor 488 conjugated goat anti-rabbit and Alexa Fluor 488 conjugated rabbit anti-mouse (both 0.5 µg/mL)) were added and incubated for 30 min at 37 °C, 5% CO_2_, followed by two washes with HBSS 1X. A fluorescent nucleic acid stain detecting double-stranded DNA in membrane disrupted cells (NETs) (Sytox Orange; 1:5000, Life Technologies), and WGA (Wheat germ agglutinin; 1 µg/mL, ThermoFisher) to detect cell membrane were added. Images (Z stack) were obtained by confocal microscopy (LSM 710, Carl Zeiss).

### Statistical analysis

The data are presented as mean ± SEM. All statistical analyses were performed using GraphPad Prism 9.1.2. Groups were compared by analysis of variance (ANOVA), followed by Tukey’s post-test for multiple comparisons. The results were considered significant if *p* values were < 0.05.

## Supplementary Information

**Additional file 1**. Individual patient characteristics and raw data for NETs release, Ang1 and calprotectin synthesis and release from human neutrophils.

## Data Availability

Not applicable.
